# Twelve Month Efficacy of Computer-Tailored Communication in Boosting Fruit and Vegetable Consumption Among Adults Aged Forty and over: A Three-Level Meta-Analysis and Systematic Review of Randomized Controlled Trials

**DOI:** 10.1016/j.advnut.2023.100150

**Published:** 2023-11-17

**Authors:** Andreja Misir, Wolfgang Viechtbauer, Hein de Vries, Ilse Mesters

**Affiliations:** 1 Department of Epidemiology, Maastricht University, Maastricht, Netherlands; 2 Department of Psychiatry and Neuropsychology, Maastricht University, Maastricht, Netherlands; 3 Department of Health Promotion, Maastricht University, Maastricht, Netherlands

**Keywords:** Computer-Tailored Communication (CTC), Fruit (mesh) and Vegetables (mesh), Intake, Diet (mesh), nutrition (mesh), Dietary Surveys (mesh), Behavior (mesh), middle age (mesh), aged (mesh), Multi-Level Analysis (mesh)

## Abstract

Computer-tailored health communication (CTC) can enhance fruit and vegetable (F&V) intake and, consequently, health by providing personalized feedback. However, few studies have examined the long-term effects of such interventions in middle-aged and older adults.

This research aimed to assess the 12-mo efficacy of CTC in promoting F&V consumption and potentially identify who among middle-aged and older adults changed their diet after the intervention. The protocol was registered at the International Prospective Register of Systematic Reviews (PROSPERO) on 2021-12-09, code CRD42022330491. The research was performed without external funding.

We searched 6 databases (MEDLINE via PubMed, EMBASE, Scopus, Web of Science Core Collection, Cochrane Library, and PsycINFO) for randomized controlled trials (RCTs) comparing CTC interventions for increasing F&V intake with usual care/no intervention control in adults aged ≥40, measured 12 mo after the pretest. The search covered the period from 1 January 1990 to 1 January 2022. We selected 16 RCTs with 25,496 baseline participants for the review systematic literature reviews (SLR) and 11 RCTs with 19 measurements for the meta-analysis (MA). We assessed risk of bias with the JBI Critical Appraisal Checklist.

The SLR revealed that at 1-y postCTC intervention, most of the treatment groups increased F&V intake more than the control groups. The overall bias in the data set was not high. The MA model on 11 RCTs revealed a significant effect size for F&V consumption in intervention groups compared with control, standardized mean difference of 0.21 (confidence interval [CI]: 0.12, 0.30), *P* = 0.0004.

The evidence suggests that CTC is a suitable strategy for public interventions aiming to increase F&V intake in adults aged ≥40. The design of CTC for public interventions should consider the process of change and stages of change addressing awareness, attitudes, self-efficacy, and social influence as promising concepts for influencing behavior change.

**Statements of Significance**

To our knowledge, this study is the first to provide a comprehensive and robust synthesis of the long-term efficacy of computer-tailored communication (CTC) interventions for increasing F&V intake in adults aged ≥40 y. Our findings have important implications for public health policy and practice, as they suggest that CTC is a feasible and efficacious way to promote 12-month sustained improvements in healthy eating habits in this population group, particularly given its relative affordability, minimal risks and ease of implementation.

## Introduction

Ensuring adequate consumption of fruits and vegetables (F&V) is crucial for maintaining a healthy diet, as it provides the body with essential nutrients such as vitamins, minerals, dietary fiber, plant sterols, flavonoids, antioxidants, and other beneficial phytochemicals [1]. A diet rich in F&V has been shown to have overall positive effects on health, improving every aspect of bodily functioning, from blood pressure to eyesight [[1], [2], [3], [4], [5], [6], [7]]. The evidence suggests that middle-aged and older adults’ health suffers when their lifestyle includes a diet with insufficient amounts of F&V [8]. Unfortunately, consumption of F&V in many regions of the world is still low [9].

These data indicate that appropriate public health initiatives to increase F&V intake among middle-aged and older adults are needed. Dietary advice has been shown to benefit from personalization [10]. Computer-based health information tailoring is a method of assessing individuals (e.g., on sociodemographic, target behavior status, and social-behavioral determinants) and selecting communication content that employs data-driven decision rules that automatically generate personalized feedback from a database of content elements [11]. Computer-Tailored Communication (CTC) has shown promise as a method for initiating improvements in people’s health behaviors. It might also encourage maintenance of diet change-improvement [[12], [13], [14], [15], [16], [17], [18], [19]].

CTC covers an array of methods that deliver individualized messages to each recipient with the aim of a larger intended communication effect than nontailored messages [12,13]. ‘Tailoring’ was first used in the 1990s, and research has shown that it helps messages reach their target more effectively than nontailored [13,20]. There are 2 classes of ‘computer tailoring’ goals: enhancing cognitive preconditions for message processing and enhancing message impact through modifying salient behavioral determinants of goal outcome [13]. It uses personalization, feedback, and content matching for message creation [13]. In the first stage of tailoring, participants self-report information on their various characteristics. In the second stage, this information is processed by a computer to tailor the message that is then delivered to the participant in the intervention [21].

To find data on the efficacy of CTC in increasing F&V intake in middle-aged and older adults, we performed a preliminary search of meta-analyses (MA) and systematic literature reviews (SLR) in Google Scholar. This search revealed that MA and/or SLR studies have been conducted on CTC, including dietary behaviors, with the most recent published in 2019 [[20], [21], [22], [23], [24], [25]]. These reviews indicated that CTC is effective in dietary behavioral change in the short term with very small to moderate effect sizes. However, existing reviews have not addressed long-term results of at least 12 mo, and none of them had focused on the somewhat older population targeting adults aged ≥ 40, although reviews have included diverse age groups. Thus, this SLR and MA aim to address these research gaps by evaluating the 12-mo efficacy of CTC in increasing F&V intake among adults aged ≥40, to identify the characteristics of adults who successfully increased their F&V intake after CTC intervention, to examine the measuring instruments used for nutritional intake (e.g., Food Frequency Questionnaire, FFQ), to evaluate the methodological quality of randomized controlled trials (RCTs) conducted on CTC interventions using the JBI Critical Appraisal Checklist, and to provide recommendations for future research.

## Methods

We developed the SLR/MA protocol following the Preferred Reporting Items for Systematic Reviews and Meta-Analysis (PRISMA) statement [26]. The protocol was registered prospectively at the International Prospective Register of Systematic Reviews (PROSPERO) under the code CRD42022330491 (9th December 2021).

## Eligibility

In the SLR, we included RCTs that tested CTC interventions for increasing F&V intake in adults with a mean age of ≥40. The studies had to compare CTC interventions with a control condition and measure F&V intake at baseline and 12 mo later (F&V intake assessed at least twice and in the same season to account for seasonal variations). We excluded reviews, case studies, case reports, observational studies, management guidelines, commentaries, or opinion papers. We also excluded studies that involved pregnant females, children, teenagers, or adults < 40 y of age and studies that used CTC interventions for behaviors other than F&V intake.

## Search Strategy

From January 1, 1990 (the decade when research on computer tailoring started) to January 1, 2022, we searched 6 databases (MEDLINE via PubMed, EMBASE, Scopus, Web of Science Core Collection, Cochrane Library, and PsycINFO) for RCTs on CTC interventions for increasing F&V intake compared with usual care/no intervention control with an adult study population of mean age ≥ 40 y with the F&V intake measured at 12 mo after the pretest.

To ensure that all the relevant articles from various sources were discovered, we checked Google Scholar, search alerts in searched databases, referenced literature, and secondary sources (e.g., citations from already identified studies).

We used the following search terms:

MeSH: “diet,” “tailored communication,” “computer-tailored,” “behavior change” OR “behaviour change” OR “diet change” AND Keywords: “vegetable intake” OR “fruit intake” AND “weight loss” OR “BMI” AND The Publication Type: “randomized controlled trial” OR “RCT.”

We initially downloaded the articles into Mendeley software, where we deleted duplicates. Then, we imported articles into Rayyan [27] to conduct independent and blinded study screening. Initially, we screened articles solely based on their title and abstract. We excluded studies if it was clear from their title and abstract that they were not eligible. Following the first screening round, we independently examined full copies of the articles for eligibility based on the inclusion and exclusion criteria described above. The blinding was removed after the first screening round. The final decision on which studies to include was made by consensus. Only the papers that answered the research question were considered.

## Quality Assessment for included studies

Two reviewers (AM and IM) separately assessed the methodological quality and potential for bias of the extracted articles, first by using the JBI Critical Appraisal Checklist [28] for RCTs. Disagreements were resolved through dialog. JBI appraisal was transferred into a generic-abbreviated assessment that was performed with the robvis [29] application. Detailed JBI appraisal forms are available upon request.

## Data analysis

We have narratively synthesized findings on the SLR regarding the theoretical base of the interventions, measurements used, or populations included [30].

We have performed the MA on 11 studies and 19 measurement entries with sufficient uniformity in the available outcome data [17, [31], [32], [33], [34], [35], [36], [37], [38], [39], [40]]. The analysis included study arms that used only CTC interventions and the corresponding control condition. Studies were required to have data on the mean number of servings of F&V or fruits and vegetables separately, as well as their standard deviation (SD) for 12-mo intake measurement. We performed (2 researchers, AM and IM) data extraction consecutively. For data from Kanera et al., 2017 [37], we contacted the authors, and the original data set was used to extract the required missing information.

For the MA, we used standardized mean differences (SMDs) corrected for their positive bias (i.e., Hedges’ g values) as the effect sizes due to the use of different instruments for measuring intake across studies (e.g., FFQs with different underlying food databases, FFQs-short versus FFQs long forms). The SMDs were calculated so that positive values indicate a higher mean F&V intake in the group receiving the CTC intervention compared with the control condition. For 10 studies [17,31,32,[34], [35], [36],[38], [39], [40]], SMD values were computed using the 12-mo intake posttest scores, and for 1 study [37] based on the baseline to 12-mo change scores, as using posttest results only would reveal an erroneous significant group difference, given there was a large baseline imbalance in vegetable intake. We followed the Cochran recommendations for MA on SMDs for combining posttest scores with change scores [41] by utilizing posttest SDs rather than change score SDs for standardizing the SMD for this study, which accurately reflected the nonsignificant group difference. For Alexander et al. (2010) [31], there was also a baseline imbalance in the 16-item FFQ in favor of control, but it did not affect the MA result whether posttest scores or change scores were used. In addition, unlike for Kanera et al. (2017) [37], for Alexander (2010) [31], no full data set was available.

Some studies allowed us to calculate multiple effect sizes: 2 studies had reported intake for F&V with more than one measuring instrument [31,34], 3 studies had reported intake for fruits separate from vegetable intake [17,32,37], and 1 had separate intake data for strata of colorectal cancer survivors and the general population with separate control for each stratum [39]. For studies that used multiple instruments, we assumed a correlation of rho=0.7 for the sampling errors of the corresponding SMD values. For studies that reported F&V intake separately, we assumed a correlation of rho=0.3 for the sampling errors. For the study that was stratified based on colorectal cancer, the sampling errors are uncorrelated due to the use of separate control groups and, hence, no overlap in participants for calculating the multiple SMD values. Based on these assumptions and the calculated sampling variances of the SMD values, we constructed an approximate variance-covariance matrix of the estimates, which was then used, together with the SMD values, as input to a 3-level meta-analysis model [[42], [43], [44]]. The model included random effects for studies at level 3 (to account for between-study heterogeneity and to allow the true effects for studies providing multiple SMD values to be correlated) and the individual estimates within studies at level 2 (to account for within-study heterogeneity).

We compared the results from the fitted model with those obtained when using cluster-robust inference methods [45]. Standardized residuals and Cook’s distances were used to identify potential outlying and/or influential studies, which were then subsequently excluded from the analysis as part of a sensitivity analysis. In addition, one study [38] did not use appropriate randomization methods, so a sensitivity analysis for this study was also conducted. To examine the data for evidence of publication bias, we used a funnel plot [46].

We analyzed data with the statistical program R ver. 4.2.1, R package metafor [42] as recommended by Pastor and Lazowski (2016) [44], robvis [29], and dmetar [47].

## Results

The search via the 6 databases yielded 1,311 publications; 30 additional articles were identified through other sources (e.g., search alerts in searched databases and referenced literature in found articles). After we removed duplicate records, we screened the title and abstract of 1128 studies, resulting in the exclusion of 1061 studies. After we applied inclusion and exclusion criteria, we selected 17 studies (16 RCTs, 2 studies from Van Keulen were part of the same Vitalum project [17,19] for the SLR, of which 11 studies with 19 entries we selected for the MA. The list of excluded studies we recorded together with the reason(s) for exclusion **(**Supplemental Table 1**)**.

## Search results

**FIGURE 1:**
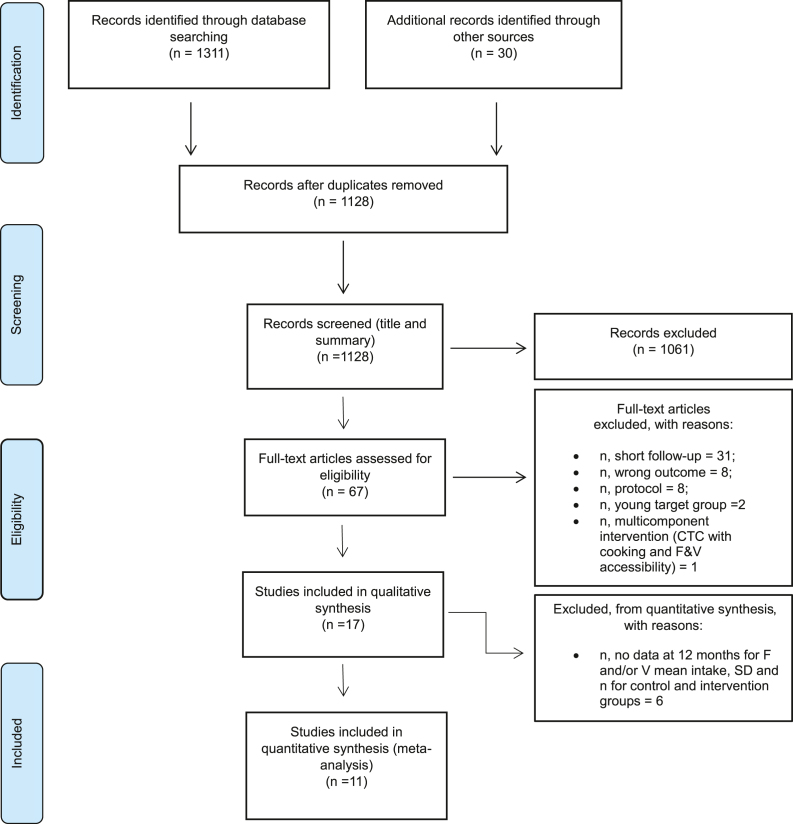
PRISMA 2009 flow diagram (Moher et al., 2009). [26]

The PRISMA flow diagram (Figure 1) displays the overall search results [26].

## Study intervention characteristics

Table 1 summarizes the characteristics of SLR studies’ interventions. These studies were published over a 21-y period (the oldest study was published in 2000 [36], and the 2 most recent ones were from 2017 [37] and 2021 [19], with the 2021 publication being related to older research).

### Number of observed behaviors and interventions

Of the 16 RCTs, 2 aimed to impact only ’one’ behavior—F&V intake [31,60]. Fourteen RCTs examined multiple behaviors, including F&V intake (Table 1). Twelve of the RCTs were part of a larger project on multiple behaviors. Four RCTs had more than 1 intervention (other interventions besides CTC, e.g., motivational interviewing) and combined interventions (Table 1). Three studies had more than 1 intervention but without combining interventions (Table 1).

**Table 1:** Study Intervention Characteristics SRL & Meta-Analysis

First author, year	Part of a Larger Project	Intervention	Targeted Health Behaviors	Number of Participants at Baseline (Number of Randomized Subjects)	Number of Arms with Control	More than One Intervention / Combined Interve-ntion	Validated FFQ or Other Measures of Diet/Recall Time for FFQ	Measu-rements	Paying Participants	Funding/Registration Trial
⊆ Alexander et al., 2010 [31]	N/A	Comparing a CTC website, a CTC website plus motivational interviewing–based counseling via e-mail, and an online untailored program.	F&V intake	2513 (2540)^3^	3	YES/YES	YES, 16-item FFQ (NCI FFQ) [48] (past month) and 2-item questionnaire (1 for total servings of fruit and one of the vegetables on a typical day) / FFQ past month	baseline, 3, 6, and 12 mo	YES	The Cancer Research Network with funding from the National Cancer Institute.
⊆ Broekhuizen et al., 2012 [32]	PRO-FIT	Comparing CTC intervention consisting of web-based lifestyle advice, face-to-face counseling, and telephone booster sessions with control.	diet, physical activity, smoking, statin therapy	340 (340)	2	NO/NO	YES, FFQ Short Dutch [49]/ FFQ typical week during past month	baseline, 12 mo	NO	Netherlands Organization for Health, Research, and Development NTR 1899
⊆ Demark-Wahnefried et al., 2007 [33]	FRESH START	Compared CTC intervention workbooks and newsletters with control intervention -standardized intervention to mailed materials.	F&V intake, fat intake, exercise	543 (543)	2	NO/NO	YES, Diet History [50]/ usual food consumption	baseline, 12 mo	YES	National Institutes of Health, the American Institute of Cancer Research, and the Susan G. Komen Foundation (W.D.-W.).
⊆ Greene et al., 2008 [34]	THE SENIOR	Comparing CTC intervention with manuals, newsletters, expert system reports, and coaching calls with a control condition.	F&V intake, exercise	1277 (1280)^3^	2	NO/NO	YES, 4 FFQ screeners (2 FFQs-9 items with portion size, FFQ 5aDay-7 items, only frequency question: ”How many servings of F&V do you usually eat each day?”) [48,51,52], for the subset of participants (*N =* 184) 24H / FFQ 5aDay-past month; 2x FFQ 9 items (based on NCI F&V screeners, but no reliable information on recall time)	baseline, 12 and 24 mo	NO	The National Institute on Aging through the National Institutes of Health.
Heimendinger et al., 2005 [60]	N/A	Comparing 4 groups: single CTC (ST) group—one tailored booklet, multiple CTC (MT) group—four CTC materials, multiple computer re-tailored (MRT) group -tailored materials with retailoring based on new information obtained at 5 mofollow-up, and a single untailored (SU) group—one untailored set of materials.	F&V intake	3402 (N/A)	4	YES/NO	YES, FFQ-7 items [53], and single-item measure / FFQ 5aDay-past month/similar to Serdula et al., 1993^,^[52], but no reliable information on recall time)	baseline, 5 and 12 mo	NO	National Cancer Institute Program
⊆ Jacobs et al., 2011 [40]	N/A	Comparing CTC intervention and individual coaching and/or web coaching intervention and control.	body weight, F&V intake, saturated fat intake, physical activity, smoking	287 (314)^3^	2	NO (but more than one dose)/NO	YES, FFQ-6 items [54] / usual intake (no reliable information on recall time)	baseline and 12 mo^1^	NO	De Onderlinge Ziekenkas (Belgium) ISRCTN23940498
Jones et al., 2003 [63]	DiSC	Comparing a CTC intervention developed from the Transtheoretical Model of Change (TTM) -Pathways to Change (PTC) and diabetes Treatment as Usual (TAU)	healthy eating, smoking	1029 (1029)	2	NO/NO	YES, FFQ (NCI Block) / usual intake or past month (no reliable information on recall time)	baseline and 12 mo	NO	Unrestricted grant from LifeScan, a Johnson and Johnson Company.
⊆ Kanera et al., 2017 [37]	N/A	Comparing a CTC fully automated web-based cancer aftercare (Cancer Aftercare Guide, KNW) and usual care (waitlist control).	vegetable intake, physical activity,	462 (518)^3^	2	NO	YES, FFQ Dutch Standard / FFQ typical week during the past month	baseline and 12 mo^2^	NO	Dutch Cancer Society NTR3375
⊆ Kramish Campbell et al., 2004 [35]	WATCH PROJECT (Wellness of African Americans through Churches)	Comparing a print and video CTC, a lay health advisor intervention, the first 2 interventions combined and control.	diet, physical activity, colorectal cancer screening	850 (N/A)	4	YES/YES	YES, FFQ- 60 items [53]/ FFQ usual diet min. 6 mo back (no reliable information on recall time)	baseline and 12 mo	NO, but paying churches for participation	American Cancer Society U.S. Department of Agriculture, and the National Institutes of Health
⊆ Kramish Campbell et al., 2009 [39]	NC STRIDES	Comparing print CTC, telephone motivational interviewing, the combination of the first 2, and control.	F&V intake, physical activity	825 (922)^3^	4	YES/YES	YES, FFQ-35 items, 2 item measure - how many servings per usual day */* FFQ past month	baseline and 12 mo	NO	National Cancer Institute, National Institutes of Health
⊆ Kristal et al., 2000 [36]	THE PEP TRIAL (The Puget Sound Eating Patterns)	Comparing CTC self-help dietary intervention and usual care.	F&V intake, fat intake	1459 (1459)	2	NO/NO	YES, FFQ-6 items, 24H [55]/ usual min. 6 mo back (no reliable information on recall time)	baseline, 3 and 12 mo	NO	National Institutes of Health
Parekh et al., 2014 [61]	10 SMALL STEPS STUDY (10SS)	Comparing CTC feedback on multiple health behaviors (diet, physical activity, alcohol, smoking) with control. Intervention and control had dual and single contact groups.	diet, physical activity, alcohol, smoking	4676 (4678)^3^	2	YES/NO	Other, intake of F&V: 0=below recommendation, 1=achieving or exceeding recommendation / not available	baseline, 3 and 12 mo	NO	BUPA Health Foundation ACTRN12611001213932
Robroek et al., 2012 [62]	NA	Comparing CTC (access to several additional website functionalities) with participants in the reference condition – a standard program consisting of a physical health check with face-to-face advice and personal feedback on a website.	F&V intake, physical activity	924 (924)	2	NO/NO	YES, FFQ-9 items Dutch [56]/ FFQ typical week during past month	baseline, 12 and 24 mo	NO	Netherlands Organization for Health Research and Development ISRCTN52854353
Schulz et al., 2014 [64]	myHealthyBehaviour	Comparing web-based simultaneous CTC, web-based sequential CTC, and control.	F&V intake, physical activity, alcohol, smoking	5055 (5390)^3^	3	YES/NO	YES, separate F and V FFQ-4 items / FFQ recall question “How many days a week do you usually eat …”, FFQ typical week during the past month	baseline, 12 and 24 mo	NO	Netherlands Organization for Health Research and Development NTR 2168
⊆ Van Keulen et al., 2011 [17]; Van Keulen et al., 2021 [19]	VITALUM	Comparing CTC letters, motivational calls, combined version of the first 2, and control.	F&V intake, physical activity	1629 (1629)	4	YES/YES	YES, FFQ-16 items [56], and the question *"How many days a week do you eat at least* *200 g of vegetables / 2 pcs of fruit?"* / FFQ typical week during the past month	baseline, 6, 11, and 16 mo	NO	Netherlands Organization for Health Research and Development NTR1068
⊆ Walker et al, 2009 [38]	WELLNESS FOR WOMEN	Comparing CTC newsletters and generic newsletters.	healthy eating, physical activity	225 (225)	2	NO/NO	YES biomarkers (nonspecific for F&V intake), semi-quantitative FFQ (HHHQ) [57], / past year at baseline, past 6 mo at 12 mo	baseline, 6 and 12 mo	NO	National Institute of Nursing Research, National Institutes of Health Nebraska Medical Center, the University of Nebraska Medical Center and The Hygienic Corporation Thera-Band® Academy

CTC-computer-tailored communication; FFQ-food frequency questionnaire; HHHQ=Health Habits and History Questionnaire [57]; ⊆ MA-included in meta-analysis; 24H-24 hour dietary recall

1 6 mo follow-up, but without F&V intake - data reported in Jacobs et al., 2011a [58];

2 6 mo follow-up reported at Kanera et al., 2016 [59];

3 some participants had lacking or inconsistent data at baseline and were not included in the final baseline data set;

Six RCTs had more than 2 arms, and the number of measurements ranged from 2 with a baseline to 4 with a baseline (Table 1).

### Instruments used for measuring nutritional intake

The most used instrument for measuring F&V intake was the FFQ (Table 1). FFQs provide information on the consumption of queried foods and beverages over the specified period. FFQs may assess total dietary intake as well as specific dietary aspects. The specific formats used are shown in Table 1 and they range from short screeners targeting only F&V to longer FFQs. Sometimes, they were combined with a targeted question on intake of F&V (“How many servings per day?” or “How many days a week do you eat at least 200g vegetables/2 pieces of fruit?”). FFQs were validated in different countries, and studies used their country-specific food tables for intake calculations. One study [33] used diet history, and one study [61] measured achieving/not achieving recommended intake with a self-administered questionnaire on health behaviors that encompassed questions on intake of F&V per day (at least 5 servings of vegetables and 2 servings of fruit per day was considered as achieving recommended intake).

The recall guideline for the length of FFQ in Dutch studies was a typical week during the past mo, based on references and additional materials (e.g., Dutch PhD database: https://www.narcis.nl/). The past month was also the most common recall length in the SLR data set (Table 1). Walker et al. (2009) [38] did not specify the recall details, such as a typical week, but required a recall of 6 mo from the last measurement baseline.

### Characteristics of meta-analyzed studies

All meta-analyzed studies had a 1-y follow-up. In some cases, there was a slightly different time frame for the 1-y follow-up, e.g., Van Keulen, 2011 [17], who started the measurement of intake in week 47 (around 11 mo after baseline). From the studies included in the meta-analysis, only 1 study [34] also reported on a 24-mo follow-up.

## Population characteristics

Table 2 summarizes the examined studies’ demographic information at baseline or for completers where the baseline was unavailable. One study specifically looked at females [38], 3 studies [32,33,62] had > 50% of females at baseline, and 6 studies had > 60% of females [31,34,35,37,60,61] at baseline.

**Table 2:** Population Characteristics

First author, year	Country	Number of Participants at Baseline or Number of Completers	Mean Age (y)	Gender	Marital Status	Education	Employment	Income	Race/Ethnicity as Defined in the Original Study	Health (main disease, BMI)
⊆ Alexander et al., 2010 [31]	US	2513	46.3y (SD 10.8)	female 69.0%	married or with a partner 72.0%	high school 9.0%, associate degree or some college 41.0%, college 26.0%, postbachelor, 24.0%	N/A	N/A	African American 24.0%, Hispanic 8.0%	N/A
⊆ Broekhuizen et al., 2012 [32]	the Netherlands	340	45.3y^1^	female 56.7%;	N/A	low=3.4%^1^, medium=60.5%^1^, control, *N =* 137: high=33.6%; intervention *N =* 163: high=38.7%	N/A	N/A	N/A	FH patients; control BMI 27.1 (SD5.3); intervention BMI 26.1 (SD4.7)
⊆ Demark-Wahnefried et al., 2007 [33]	US	543	57.0y (SD 10.8)	female 56,4%	N/A	<high school 12.0%, some college or associate 30.0%, college graduate/postgraduate 58.0%	N/A	N/A	White 83.0%, Black 13.0%, Other 4.0%	newly diagnosed locoregional breast or prostate cancer, 64% overweight/obese
⊆ Greene et al., 2008 [34]	US	completers 834	74.7y(SD 6.4)	female 72.9%	married-cohabit 47.7%, widowed 37.9%, other 14.4%	<12 y 19.5%, high school 38.5%, some college 22.5%, college 19.9%	N/A	N/A	Caucasian 79.5%, Hispanic-Portuguese 13.2%, Other 7.3%	BMI 27.3(SD4.9)
Heimendinger et al., 2005 [60]	US	3,402	46.3y^1^	female 82.0%	N/A	high school or less 28.0%, some college/college grad 55.0%, postcollege17.0%	N/A	N/A	African American 15.0%, Hispanic 10.0%, nonHispanic White 63.0%, Asian/Pacific 6.0%, other 6.0%	N/A
⊆ Jacobs et al., 2011 [40]	Belgium	287	40.5y (SD 10.6)	female 33.4%	N/A	Master’s in law 100%	self-employed layers 100%	N/A	N/A	cardiovascular disease risk: low 69.7%, average 10.8%, high 8.4%, unknown 11.2%; mean BMI 25.1 (SD 4.1)
Jones et al., 2003 [63]	Canada	1,029	±54.8y	female 47.6%	N/A	N/A	N/A	N/A	N/A	diabetes patients (T2D, T1D), mean BMI 31.8
Kanera et al., 2017 [37]	The Netherlands	416	± 55.9y	female 79.9%^1^	with partner 81.6%^1^	low education 37.5%, medium education 31.6%, high education 31.0%^1^	employed 50.5%^1^	above average income 49.9%, average income 35.1%, below average income 15.2^1^	N/A	cancer survivors, BMI 26.3
⊆ Kramish Campbell et al., 2004 [35]	US	completers 587	52.0y	female 74.3%^1^	58.0% married or living with a partner	±25.0% some education beyond high school	N/A	N/A	African American 99.0%	±40.0% with BMI 30.0 ≥
⊆ Kramish Campbell et al., 2009 [39]	US	735 completers	66.5y	female 49.4%	married/partnered 67.5%, divorced/widowed 29.4%, never married 3.2% (calculated for nonmissing values)	≤high school 42.7%, some college 28.3%, ≥ college degree 29.1% (calculated for nonmissing values)	employed 37.5%, retired/unemployed 62.5% (calculated for nonmissing values)	<$30,000 45.0%, > $30,000 55.0%(calculated for nonmissing values)	African American 35.4%, White 64.6%	CRC survivors and nonCRC individuals; mean BMI 29.1 (SD5.7)
⊆ Kristal et al., 2000 [36]	US	1,459	44.9y (SD 14.9)	female 49.1	*N =* 1205 living alone 11.5%, living with other adults 50.3%, living with children 38.2%	N/A	N/A	$35,000-$69,000 49.1%	White 85.9%, Black 4.5%, Asian 5.8%, Hispanic 3.0%, Other 0.8%	BMI 26.5 (SD 5)
Parekh et al., 2014 [49]	Australia	completers 2873-2863	*N =* 2867: 46.9y (CI 46.5-47.3)	*N =* 2872: female 69.2%	*N =* 2865: married 71.7%, single 28.3%	*N =* 2863: high school and below 41.3%, university 58.7%	*N =* 2865: employed 65.2%, unemployed 34.8%	*N =* 2867: Area of Residence: affluent 44.0%, disadvantaged 31.6%, most disadvantaged 24.4%	N/A	N/A
Robroek et al., 2012 [62]	The Netherlands	924	42.0y (range 20.0-63.0)	female 51.0%	unmarried/not cohabited 24.0%^1^	lower 22.4%^1^, intermediate 33.1%^1^, higher 45.2%^1^	employed 100%	N/A	nonDutch 16.5%^1^	obesity 9.0%^1^, elevated blood pressure 32.0%^1^, elevated cholesterol level 46.1%^1^, poor or moderate VO2 max 38.7%^1^
Schulz et al., 2014 [64]	The Netherlands	5,390	44.2y (SD 12.7) (range 19-65)	female 47.4%	relationship 76.1%, single 23.9%	low 10.4%, medium 47.1%, high 42.6%	job 76.2%, no job 23.8%	<1750 €/mo 23.4%, 1751-3050€/mo 34.0%, >3051€/mo 28.1%, not reported 14.5%	Dutch residents 95.1%, Other 5.0%	diabetes 2.9%, high blood pressure 12.4%, mean BMI 25.2 (SD 4.0)
⊆ Van Keulen et al., 2011 [17]; Van Keulen et al., 2021 [19]	The Netherlands	1,629	57.2y (SD 7.1)	female 45%	married or living together 79.8%^1^	low 54%, intermediate 23%, high 23%	N/A	N/A	Dutch residents 95.0%	hypertensive 52%, BMI <18 1%, 18.5-25 31%, 25-30 45%, ≥30 22%
⊆ Walker et al, 2009 [38]	US	225	57.8y (age range 50.0-69.0)^1^	female 100%	married 70.2%, not married 16.4% (missing data, totals do not add)^1^	some high school or less 3.6%, high school 21.3%, some college 39.6%, college graduate or higher 34.7%^1^	employed full-time 46.7%, employed part-time 18.2%, not employed outside home 34.2%^1^	±62% in the range 20-59K^1^	>94.0% White nonHispanic^1^	mean BMI 30.2^1^

1 mean calculated from the available data in the study, CRC-colorectal cancer, FH-familiar hypercholesterolemia, ⊆ MA-included in meta-analysis

Five studies included participants with pre-existing conditions such as diabetes [63], cancer [33], cancer survivors [37,39], and familial hypercholesterolemia [32]. One study was performed on a 99% African American population in the United States [35]. These studies were conducted in various countries, with 8 being done in the United States [31,[33], [34], [35], [36],^38^,^39^,^60^], 6 in the Netherlands [17,19,32,37,62,64] (one RCT resulted in 2 papers), 1 in Belgium [40], 1 in Canada [63], and 1 in Australia [61].

## Behavior change approaches

Theoretical models and concepts used for tailoring have been reported in all SLR studies **(**Table 3**)**. Robroek et al., 2012 [62] only provided information on the measured social-cognitive variables (concepts) without specifying the model.

**Table 3:** Studies and Theoretical Models Used

First author, year	Theoretical Model for Tailoring	Concepts
Alexander et al., 2010 [31]	SCT, TTM, HBM	motivation to change, barriers to change, cues to action
Broekhuizen et al., 2012 [32]	I-Change Model	action/behavior, motivation, awareness
Demark-Wahnefried et al., 2007 [33]	SCT, TTM	stage of readiness, cues to action, skills development, self-efficacy,
Greene et al., 2008 [34]	TTM	stage of change, process of change, self-efficacy
Heimendinger et al., 2005 [60]	SCT, TTM, HBM	stage of change, barriers to change, goal setting, self-efficacy, perceived susceptibility
Jacobs et al., 2011 [40]	TPB, SDT	intentions, perceived behavioral control, attitudes, subjective norms
Jones et al., 2003 [63]	TTM	stage of change
Kanera et al., 2017 [37]	TPB, SRT, I-Change	barriers to change, action planning, goal setting, self-efficacy, beliefs
Kramish Campbell et al., 2004 [35]	SCT, TTM, HBM, SSM	current behavior, stage of readiness to change, barriers to change, social support, beliefs,
Kramish Campbell et al., 2009 [39]	SCT, TTM	stage of readiness to change, barriers to change, motivation, social support, self-efficacy, knowledge/awareness
Kristal et al., 2000 [36]	SLT, TTM, DIM	stage of readiness to change, self-efficacy, knowledge, attitudes, motivation, skills
Parekh et al., 2014 [61]	Modified Events of Instruction Framework [65], Elaboration Likelihood Model [66]	gain attention, present stimuli, provide guidance, elicit performance, and provide feedback - (basic concepts framework further populated with strategies based on HBM, SCT, DT)
Robroek et al., 2012 [62]	N/A	intention to change, perceived barriers, self-efficacy
Schulz et al., 2014 [64]	I-Change Model	current behavior, intention to change, preparatory plans, coping plans, social influence, self-efficacy, attitude
Van Keulen et al., 2011 [17]; Van Keulen et al., 2021 [19]	I-Change Model, CT	current behavior, stage of change, action plans, self-efficacy, attitude, expectations, awareness, age, gender, social norms, and social modeling
Walker et al, 2009 [38]	HPM (based on SCT)	self-efficacy, barriers, perceived benefits, interpersonal influences

CT=Control Theory, DIM=Diet Individuation Model, DT=Diffusion of Innovation Theory, HBM=Health Belief Model, HPM= Health Promotion Model, N/A=not available, SCT=Social-Cognitive Theory, SDT=Self-Determination Theory, SLT=Social Learning Theory, SRT=Self-Regulation Theory, SSM=Social Support Models, TTM=Transtheoretical Model, TPB=Theory of Planned Behavior

## Intervention effects and sustained outcomes

SRL on 16 RCTs found that after 1 y, the treatment groups in most of these studies had a greater intake of fruits and/or vegetables compared with the control groups, though the degree of improvement varied **(**Table 4**)**.

**Table 4:** Key Findings of Analyzed Studies

First author, year	Population	Intervention Characteristics (arms and measuring instruments)	Key Findings at 12 Mo (intervention vs. control on posttest and/or change scores)
⊆ Alexander et al., 2010 [31]	• US, 69% female, mean age=46.3y	• control, web-based CTC, CTC intervention with motivational interviewing–based counseling via e-mail • FFQ-16 items and 2 items measure	• FFQ-16 items showed an average F&V svg. increase of more than 2 svg. across all study arms with a statistically significant increase from baseline (+2.8svg. *P <* 0.05) compared to control only in CTC plus motivational interviewing–based counseling via e-mail. The intervention vs. control difference in the final measurement was only +0.15 svg. • 2 items measure showed average F&V svg. increase by more than 2 svg. across all study arms, with an increase of +2.55 svg. from baseline among participants of CTC and CTC plus motivational interviewing–based counseling via e-mail (*P <* 0.05 and 0.04 respectively when compared to the control group). The intervention vs. control difference in the final measurement was only +0.14 svg.
⊆ Broekhuizen et al., 2012 [32]	• The Netherlands, 56.7% female, mean age≈45y, FH patients	• control, web-based CTC lifestyle advice combined with face-to-face counseling complemented with telephone booster sessions • FFQ Dutch	• In both arms, nonsignificant improvements were found in all lifestyle behaviors.
⊆ Demark-Wahnefried et al., 2007 [33]	• US, 56.4% female, mean age=57y, newly diagnosed cancer	• control, CTC intervention • Diet History	• Diet History showed significantly improved lifestyle behaviors (p <.05) in both arms with significantly greater gains in the intervention vs. the control group. F&V intake per day increased in CTC intervention (+1.1 svg.) from baseline and had + 0.6 svg. the mean difference with control (p <0.01).
⊆ Greene et al., 2008 [34]	• US, 72.9% female, mean age=74.73y	• control, CTC intervention • 4 FFQ screeners, only for a smaller group of participants 24H	• FFQ screeners (single-item measure showed some difference at 12 mo, but not at 24 mo) showed increased intake compared to the control group (+0.5 svg. to +1.25 svg. up to 24 mo of intervention).
Heimendinger et al., 2005 [60]	• US, 82% female, mean age=46.3y	• control, single CTC, multiple CTC, multiple computer re-tailored intervention • FFQ-7 items and a single-item measure	• FFQ-7 items showed significant mean F&V svg. differences between control (5.07 svg.) vs. multiple CTC (5.64 svg., p = 0.002) and control vs. multiple computer re-tailored intervention (5.71 svg., p < 0.001).
⊆ Jacobs et al., 2011 [40]	• Belgium, 33.4% female, mean age=40.5, 100% Master’s in Law	• control, CTC intervention • short FFQ	• Short FFQ showed that the control increased F&V svg. per day by 16.62% and the intervention by 23.93% from baseline. The difference was not statistically significant. • Short FFQ showed that there was no statistically significant difference in percentage change from baseline in recommended F&V intake.
Jones et al., 2003 [63]	• Canada, 47.6% female, mean age≈54.8y, diabetes	• control, CTC intervention (PTC) • FFQ	• FFQ showed that the intervention group had significantly increased intake of fruit svg. (1.89 svg. PTC vs. 1.68 svg. control, *P <* 0.016), and vegetable svg. (2.24 svg. PTC vs. 2.06 svg. control, *P <* 0.011) compared to the control.
⊆ Kanera et al., 2017 [37]	• The Netherlands, 79.9% female, mean age≈55.9y, cancer survivors	• control, CTC intervention • FFQ Dutch	• No significant effects on change from baseline when intervention is compared with control.
⊆ Kramish Campbell et al., 2004 [35]	• US, 74.3% female, mean age=52y, 99% African American	• control, CTC print and video, lay health advisor intervention, previous 2 interventions combined • FFQ	• FFQ showed that the CTC print and video group had significantly (*P <* 0.05) improved F&V consumption (+0.6 svg.) compared to baseline and had higher F&V intake (+0.5 svg.) compared to control.
⊆ Kramish Campbell et al., 2009 [39]	• US, 49.4% female, mean age=66.5y, one group colorectal cancer (CRC) survivors	• control, print CTC intervention, telephone motivational interviewing, previous 2 interventions combined • FFQ-35 items, 2 item measure, biomarkers	• FFQ-35 items showed a significant increase in F&V consumption (+1 svg.) for the combined intervention group in the entire sample (p < 0.05) compared to the control. • No significant effect for print CTC only, although it had the greatest increase in consumption among CRC survivors (+1 svg.).
⊆ Kristal et al., 2000 [36]	• US, 49.1% female, mean age=44.9y	• control, CTC intervention • FFQ-6 items (approach from 5 a day for better health program) for F&V, 24H	• FFQ-6 items showed the adjusted intervention effect +0.46 svg. ± 0.1SE (*P <* 0.001).
Parekh et al., 2014 [61]	• Australia, 69.2% female, mean age=46.9y	• control (dual and single contact), CTC feedback (dual and single contact) • 0 and 1 scores representing below and achieving/exceeding recommendations respectively	• Statistically significant OR for comparing intervention to control groups improvements in achieving recommended F&V intake was observed in both intervention groups (single contact: 1.22, CI:10.16-1.41, *P <* 0.001; double contact: 1.37, CI:1.18-1.59, *P <* 0.00). The increase in adherence to guidelines for F&V intake was considerable, especially in the dual contact group (+9.23%).
Robroek et al., 2012 [62]	• The Netherlands, 51.0% female, mean ageV=42y	• control, CTC intervention • FFQ-9 items, Dutch	• FFQ-9 items showed that there were improvements in vegetable intake (OR: 1,36, 95% CI: 1,05-1,97) 1 y after baseline and (OR:1,43, 95% CI: 1,05-1,97) 2 y after baseline. • FFQ-9 items showed that participants in the intervention condition not meeting the guidelines for fruit at baseline were more likely to meet the guidelines at 12 mo compared to control (OR: 2.03, CI: 1.20-3.44). Nonsignificant effects were found for meeting guidelines on fruit or vegetable intake at 24 mo.
Schulz et al., 2014 [64]	• The Netherlands, 47.4% female, mean age=44.2	• control, web-based simultaneous CTC and sequential CTC intervention • FFQ-4 items screeners separate for fruit and vegetables	• FFQ-4 items showed that simultaneous CTC was nonsignificantly more effective in increasing fruit intake at 12 and 24 mo compared to the control. Both interventions were effective in promoting favorable lifestyle changes.
⊆ Van Keulen et al., 2011 [17]; Van Keulen et al., 2021 [19]	• The Netherlands, 45% female, mean age=57.2y	• control, CTC letters, motivational calls, previous 2 interventions combined • FFQ - 16 items and question about F&V intake	• FFQ-16 items showed that all 3 intervention groups were equally and significantly more effective (with some differences in favor of CTC) than the control group in increasing intake of fruit (svg./d) and of vegetables (g/d) from baseline. Effect sizes (Cohen’s d) ranged from 0.15 to 0.18. • FFQ-16 items showed that CTC group was more likely to adhere to F&V consumption guideline than control or combined group (CTC vs. control P < 0.001, average for 3 time points).
⊆ Walker et al, 2009 [38]	• US, 100% female, mean age=57.8y	• control, CTC intervention • FFQ	• FFQ showed that the CTC group significantly increased F&V svg. (+0,92 svg.) from baseline to 12 mo, unlike the control that had after initial improvement at 6 mo dropped to baseline at 12-mo measurement. The intervention vs. control difference in the final measurement was even greater +1.25 svg.

CTC, computer-tailored communication, CI, confidence interval, FFQ, food frequency questionnaire, OR , odds ratio, PTC, “Pathways to Change” intervention, svg, servings, ⊆ MA-included in meta-analysis, 24H, 24 hour dietary recall

Studies conducted in the Netherlands [17,19,32,37, 62,64] and Canada [63] report findings on F&V intake separately, whereas studies performed in the United States [31,[33], [34], [35], [36],^38^,^39^,^60^], Australia [61], and Belgium [40] report findings on F&V intake together.

## Meta-Analysis

We performed a 3-level MA to assess the 12-mo efficacy of CTC when it comes to increasing F&V intake in adults aged ≥ 40. The pooled SMD based on the 3-level MA model was SMD = 0.21 (CI: 0.12-0.30), *P* = 0.0004. The estimated variance components were τ^2^Level_3_ = 0.0088 for the between-study heterogeneity and τ^2^Level_2_ = 0.0021 for the within-study heterogeneity. This resulted in I^2^Level_3_ = 49.09% of the total variation, which can be attributed to between-study heterogeneity, and I^2^Level_2_ = 11.88%, which can be attributed to within-study heterogeneity.

**FIGURE 2:**
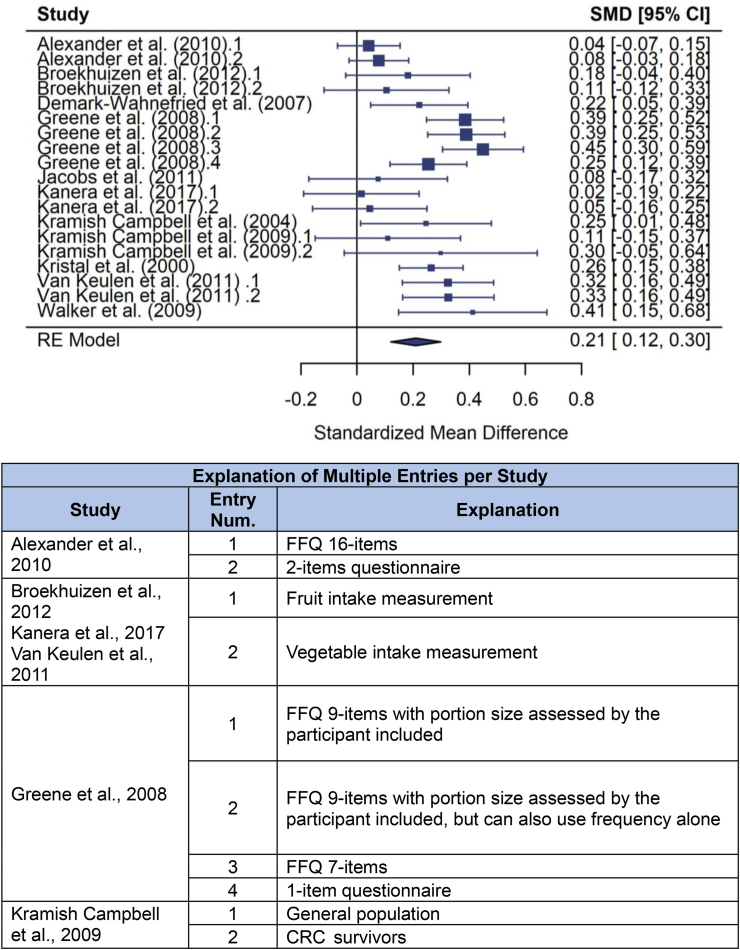
The three-level meta-analysis forest plot with the explanation table.

**FIGURE 3:**
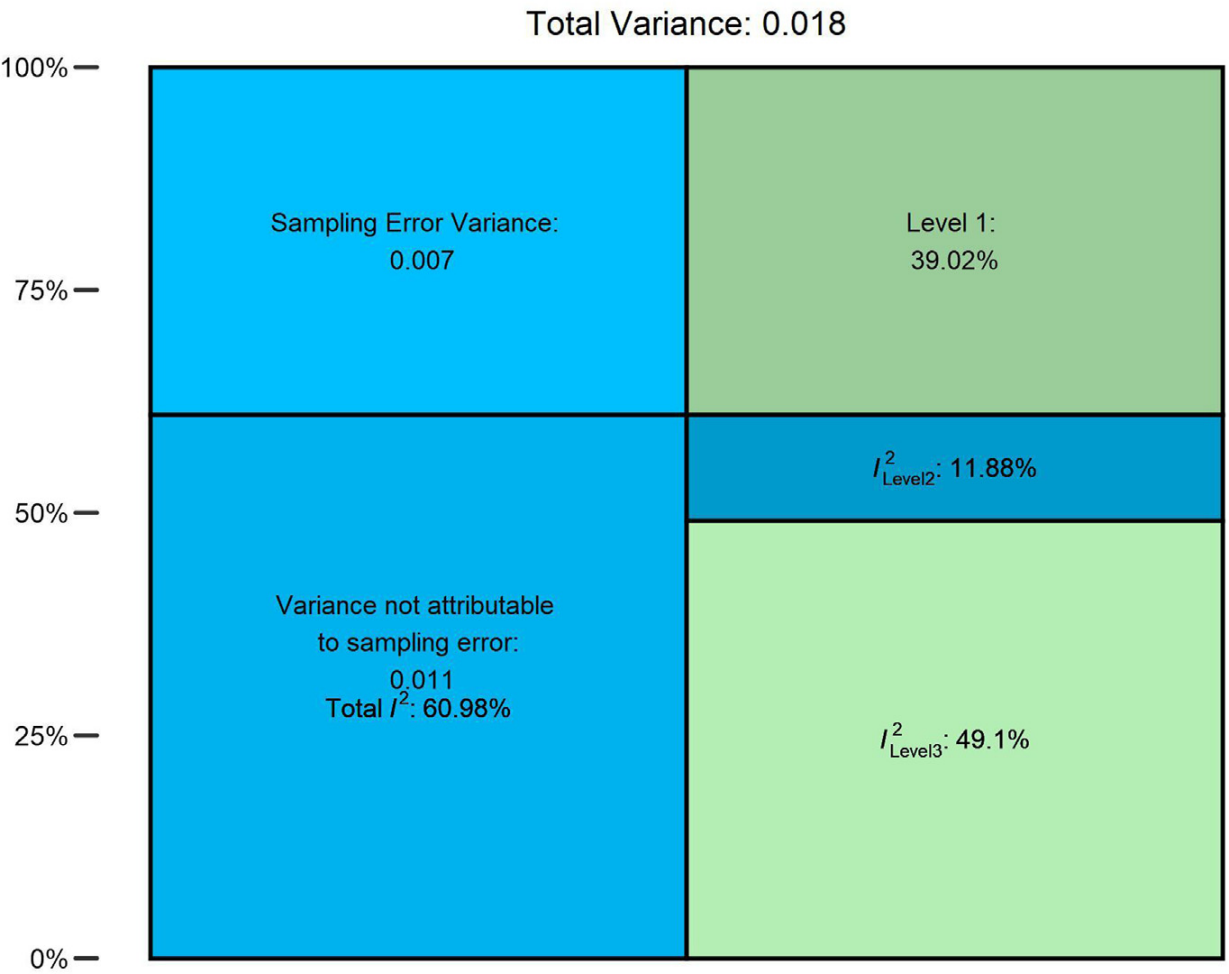
Graphic representation of the variance and heterogeneity.

Using cluster-robust inference methods did not yield noteworthy differences in results compared to the fitted model. Relative to the rest of the studies, Cook’s distance was relatively large for Walker et al. (2009) [38], but a sensitivity analysis excluding this study did not yield any relevant differences in terms of the pooled effect, confidence interval, or amount of heterogeneity. The MA is summarized in the forest plot (Figure 2), and the heterogeneity split is in Figure 3.

**FIGURE 4:**
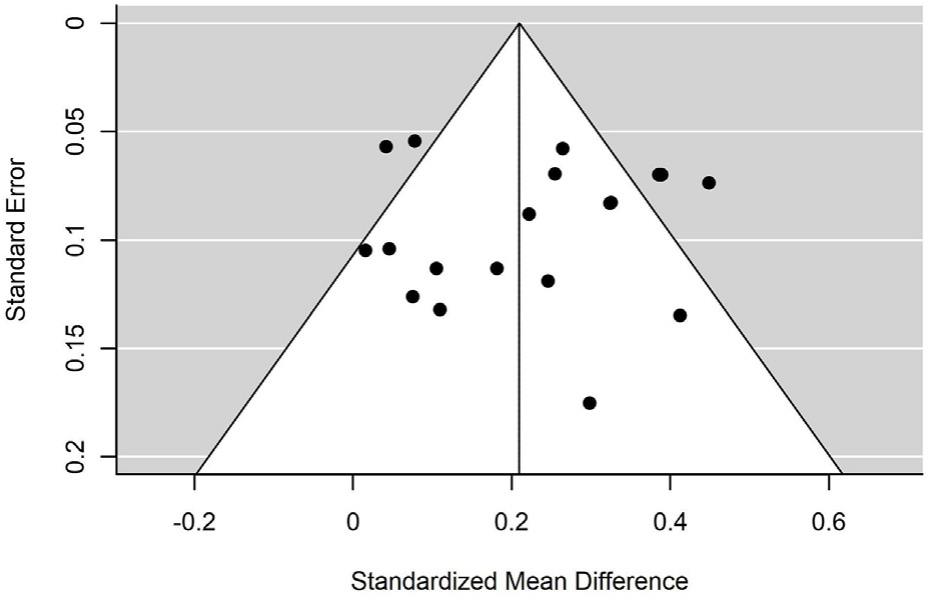
Funnel plot.

The funnel plot (Figure 4) did not show any apparent evidence for publication bias.

## Risk of bias

**FIGURE 5:**
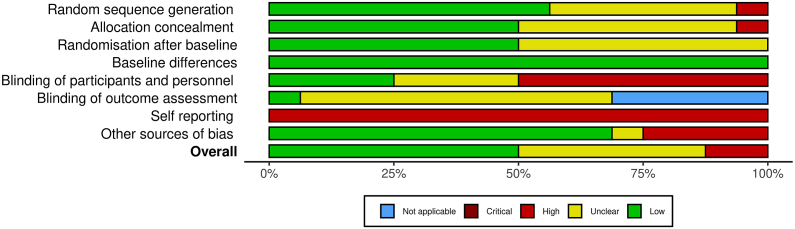
Overall, bias in the SLR data set entailing 16 studies.

The main study characteristics that we analyzed in assessing study bias were the randomization procedure and its success; blinding of participants, personnel, and outcome assessment; self-reporting; and other sources of bias (attrition, data analysis). Figure 5 shows a summary of study quality components used for assessing study bias (individual study bias is in Supplemental Figure 1).

In 6 studies, it was not defined how randomization was performed [31,[34], [35], [36],^39^,^60^]. Three studies [32,35,62] used cluster randomization, with authors accounting for cluster randomization in their statistical analysis. Walker et al. (2009) [38]] did not use proper cluster randomization and did not account for that in the analysis.

At baseline, there were group imbalances: in Alexander et al. (2010) [31] the control group ate more F&V according to the 16 items FFQ (statistically significant); in Kanera et al. (2017) [37] the intervention group ate more vegetables (*P* = 0.015); in Robroek et al. (2012) [62] the intervention group had more participants who ate enough fruit (*P* < 0.05); in Schulz et al. (2014) [64] the control group was older (*P* = 0.03) than the sequential group, experimental condition had more heart attacks (*P* = 0.01) and less high blood pressure (*P* = 0.002); in Broekhuizen et al. (2012) [32] there were BMI differences (β=-1.1; CI: -2.17, -0.04). All 5 studies were corrected for these imbalances in the analysis.

The studies used 2 main types of delivery: digital (web-based) and analog (written, phone, or face-to-face). Participants were not blinded, but mostly, digital delivery was linked to digital outcome assessment without human contact **(**Supplemental Table 2**)**. Seven studies [31,34,36,39,60,61,63] did not report allocation concealment*.* Eight RCTs were randomized after the baseline [17,[31], [32], [33],^35^,^37^,^60^,^61^], whereas for 8 RCTs there was no information on the timing of the randomization [34,36,[38], [39], [40],[50], [51], [52]] (Supplemental Table 2, Supplemental Figure 1).

Attrition rates were reported in all studies and varied from 5% [32] to 60.6% [64] at 12 mo from baseline with a mean of 23.6% (median *=* 23.4%). Differential drop-out rates with very low and low overall attrition from > 6% to 17.5% were reported by Demark et al. (2007) [33] and Kanera et al. (2017), [37] respectively. Heimendinger et al. (2005) [60] reported that the systematic loss to follow-up did not affect the composition of the experimental conditions at 12 mo with no significant differences for any of the variables or the baseline estimate for F&V consumption. Van Keulen et al. (2011) [17] report differential drop-out between groups (higher in intervention group) and education levels (higher among lower educational participants), whereas Robroek et al. (2012) [62] report more drop-out in the intervention group in the first follow-up, but they accounted for that in the analysis.

Four studies [31,35,36,39] used only a per protocol (PP) analysis. For one study [60], it is unclear whether they used a PP or intention to treat (ITT) analysis. The treatment of missing data in ITT was mostly properly reported and performed.

Measuring diet was based on self-reported tools. In 2 studies, participants were compensated for their participation [31,33], and 1 study paid churches for participating with their participants [35].

## Discussion

This SRL on 16 RCTs and MA on 11 RCTs shows that an improvement in CTC in F&V intake may last for at least 1 y for middle-aged and older adults. Furthermore, in more than half of the meta-analyzed studies, the CTC treatment group outperformed the control.

## SLR

The analysis of 16 RCTs revealed that 1 y postbaseline in 10 studies [17,31,[33], [34], [35], [36],^38^,^60^,^61^,^63^] CTC intervention groups increased consumption of F&V significantly compared with the control groups (Alexander et al. 2010 [31] solely for the 2-item measure) and in 3 studies [32,40,64] there were nonsignificant improvements. Approaches that integrated a tailored intervention with other strategies, such as motivational interviewing [17,31,39], also yielded positive outcomes 12 mo postintervention.

Nine studies [17,31,34,36,38,[60], [61], [62],^64^] included in this SLR were longitudinal, collecting data at various time points ranging from baseline to mostly 12 mo (3 studies [34,62,64] up to 24 mo). Out of the 3 studies [34,62,64] in SLR that followed participants for 2 y, 1 [34] reported a significant increase, and 1 [64] reported a nonsignificant increase in F&V consumption.

F&V intake was reported separately in the Netherlands [17,19,32,37,62,64] and Canada [63], whereas in the United States [31,[33], [34], [35], [36],^38^,^39^,^60^], Belgium [40]’ and Australia [61], combined intake was reported. However, it is more advisable to report these 2 behaviors (fruit intake and vegetable intake) separately in behavior change research because they may differ in their determinants*,* intake results, and impact on intake maintenance.

Out of 7 studies that had either all female [38] or predominantly female (over 60%) population [31,34,35,37,60,61], 6 [31,34,35,38,60,61] successfully sustained improvement in intake at 12 mo (SLR data set) (Alexander et al.,2010 [31] only for 2 item measure). This could indicate that middle-aged and older females are a successful group in sustaining their F&V intake postintervention, which corresponds to previous research on dietary behaviors finding that females are more inclined to be motivated to higher intakes of F&V than males [67,68]. Additionally, the limited number of studies [32,33,37,39,63] that involved participants with different underlying diseases did not allow for a clear assessment of how lifestyle behavior interventions might affect these populations.

CTC interventions in this SLR varied, and each research had some unique characteristics (Table 1, Table 2, Table 3) in terms of, for instance, the mode of communication (from letters to computer screens) or the country of study (United States, Netherlands, Belgium, Canada, and Australia), or in health status of participants, but the theoretical framework for the CTC method was very similar among studies and should serve as the primary guide for future research. This SRL showed that TTM, I-Change, and SCT were used most often in the past 21 y in theory-based CTC for diet change. As I-Change encompasses elements of both TTM and SCT, it can be concluded that the fundamental theoretical basis for all analyzed studies was comparable. Most analyzed studies used a combination of process of change and stages of change addressing attitudes, self-efficacy (or behavioral control), and social influence (support, pressure, and modeling) as advised by Noar et al. (2007) [20], who found these concepts to result in a larger behavioral impact.

Whereas increasing F&V intake is beneficial for health, it is important to reach the recommended intake levels for F&V. Only 4 studies [19,40,61,62] have reported intervention results for F&V intake related to increasing adherence to nutritional recommendations that were in effect in the country of research at the time of the study. Of these, 3 [19,61,62] showed statistically significant improvement in the intervention group in reaching the recommended intake levels at 12 mo follow-up. Researchers and policymakers are encouraged to monitor adherence to these guidelines in all upcoming research studies and prioritize the implementation of cost-effective interventions that promote adherence to these guidelines.

Although the cost of CTC interventions was not the primary focus of this SLR, it is a crucial consideration for interventions aimed at changing behavior. In our data set, few studies mention the cost-effectiveness [36,39,60,62] or have a separate, related study on this topic [69,70]. All except Robroek et al. (2012) [62] find CTC cost-effective and recommendable for use. Given the practical significance of cost-effectiveness for applying interventions in the real world, it is advisable that this topic be covered in any upcoming CTC research.

## Meta-analysis

MA on 11 studies resulted in a standardized effect size of 0.21 (*P* = 0.0004) that, according to Cohen [71], corresponds to a small intervention effect. However, determining whether an effect is small, medium, or large should be based on the findings of previous studies in the relevant field. A 1-y postintervention effect size of 0.21 corresponds to an effect size that is commonly found in psychology (behavioral research), which is the field to which CTC interventions belong. This effect size indicates that the implementation of these interventions can have a significant impact on public health when widely adopted [64,72,73]. Its clinical relevance becomes apparent when considering certain critical factors: affordability, minimal associated risks, and broad implementability, all characteristics that align with CTC interventions [72,73]. What demands even greater emphasis, however, is the revealed enduring impact of CTC interventions. A small but enduring effect size may possess more profound clinical significance than a larger effect that merely produces short-lived results.

The result from this MA corresponds to or is slightly better than the results of MAs on diverse CTC interventions (exercise, smoking, alcoholism, cancer screening) in various age groups [20, 72, 73]. This research focused on middle-aged and older individuals and found an overall slightly better MA result than previous research performed on more heterogeneous age groups [20, 72, 73], which could also indicate that middle-aged and older adults in CTC intervention are more likely to increase their F&V intake and consolidate this change.

## Potential for bias

Out of the 16 RCTs in this review, 12 used individual randomization [17,31,33,34,36,37,39,40,60,61,63,64], 3 [32,35,62] used cluster randomization, and 1 [38] used quasi-cluster randomization. The cluster-randomized trials adjusted their analysis accordingly. Walker et al. (2009) [38] used quasi-cluster randomization, so the study might suffer from confounding and selection bias [74]. Nevertheless, the choice of methodology can be justified by the type of research that was performed and their goals— researching diet change in hard-to-reach, older, rural females in the Midwestern United States and controling for spillover between intervention and control that can happen within a village. Thus, they used 2 demographically similar villages randomly sampled for intervention and control to get as much as possible a “representative” sample for the population they set to investigate. In addition, this study was identified as a potential outlier in MA dataset, but a sensitivity analysis did not yield any noteworthy differences in the results.

In the study by Alexander et al. (2010) [31], the tailored behavioral intervention group had lower F&V intake at baseline than the control group, measured by a 16-item FFQ. This could have biased the results in favor of the control group. Kanera et al. (2017) [37] had the opposite situation from Alexander et al. (2010) [31] and in Robroek et al. (2012) [62] the intervention group had more participants who ate enough fruit. All 3 authors reported adjustment for baseline differences.

In 8 studies [17,32,33,35,37,40,62,64], allocation treatment was ideally concealed during recruitment, and in 8 studies [17,[31], [32], [33],^35^,^37^,^60^,^61^], pretest measurement randomization was performed after baseline to prevent selection bias and ensure a balance between groups.

Blinding participants was not always possible in some of the interventions due to the nature of the study design, which is not uncommon in nutritional interventions [75]. When participants cannot be blinded, blinding care providers and assessors is important [76]. In this SLR/MA, the CTC interventions had no intervention providers, and assessments were mostly filled out at home without assessors (only self-reporting).

All studies used self-reporting tools for measuring F&V intake, with FFQs used most often. These FFQs were designed to measure intake for the typical week over the course of the past month, which is a good balance between FFQ’s aim to rely on a longer recall period (from 1 wk to as long as 1 y) to capture foods that are not consumed every day and recall bias that may increase with longer periods of recall [77,78]. Information on psychometric qualities was often limited to claims that an instrument had been validated or was otherwise reliable.

Many of the studies utilized ITT analysis, and all these studies, except for Jones et al. (2003) [63], properly reported and addressed missing data. Attrition was reported in all studies, with the mean attrition rate being 23.6%. Although drop-out > 20% may affect validity, it should be considered that in this SLR/MA studies had a 12-mo follow-up, mostly used ITT to reduce attrition bias, and did power calculations to account for drop-out and maintain power. However, Schultz et al. (2014) [64] had an attrition rate of > 50% at 12 mo, but this is to be expected for a web-based intervention [79]. In this SLR, 5 studies [17,33,37,60,62] indicated differential drop-out between control and intervention conditions, but they used ITT and mostly had an acceptable overall drop-out rate (∼20%). In addition, Crutzen et al. (2013) [80] claim that there is an indication that slightly higher attrition rates are often seen in intervention groups compared with control, which attenuates the observed effect.

Overall bias in the presented SLR data set was not high, despite that nonblinding of participants produced a high bias score, but this is common in nonpharmacological interventions [81]. Most studies were funded by national foundations and governmental agencies, which is expected to reduce risk of conflict of interest that can influence the design of the study, the interpretation of the results, and the publication of the findings.

Analysis of bias found gaps in the reported methodological details of reviewed studies, which could be avoided by adhering to CONSORT and TREND guidelines for randomized and nonrandomized studies, respectively. In this analysis, it has been acknowledged that the absence of information on a certain procedure in a published study does not necessarily imply that the procedure was not done [[82], [83], [84]].

## Strengths and limitations

The studies analyzed have limitations as they rely on self-reported dietary information, which is common in nutritional research [75]. Furthermore^,^ 1 study [38] did not conduct proper cluster randomization; 1 study [37] had more vegetable servings in the intervention group at baseline; and 1 study [31] had fewer F&V servings in the intervention group at baseline (leading to less room for improvement or vice versa, respectively). In addition, some studies have limited external validity: 4 used PP analysis (risk of overestimation of results) [31,35,36,39], 6 entailed predominantly—> 60% [31,34,35,37,60,61] or all females [38] (females can be more health driven when it comes to diet, so also show a better overall result) [67,68] and 5 analyzed participants with underlying diseases [32,33,37,39,63].

Although the narrow selection criteria for the systematic review allowed for easier comparison and greater confidence in the results, it was still difficult to ascertain specific characteristics of individuals, such as race or ethnicity, marital status, and employment status, due to numerous confounding factors. Also, only 4 studies looked at change in reaching nutritional recommendations as supposed to just increase in F&V intake [[19], [40], [61], [62]], and only 5 studies looked at patients with different underlying diseases [32,33,37,39,63]. No further analyses could be conducted on these small samples.

This research strength is its unique focus on F&V intake at 12 mo after CTC behavioral lifestyle intervention. Additionally, this is currently the most comprehensive SLR on CTC interventions and f F&V intake in the past 21 y.

Another strength is the use of state-of-the-art methods in the MA, which allowed for the inclusion of dependent estimates with proper adjustment for the correlation between measurements. However, a limitation is that only 11 studies were meta-analyzed, which excludes the possibility of subgroup analyses and limits the generalizability of the findings [43].

## Conclusion

This review shows that CTC can help middle-aged and older adults sustain their increased F&V intake 1 y after the intervention. Therefore, CTC is a suitable strategy for public interventions that aim to increase F&V intake in adults aged ≥ 40. The design of CTC for public interventions should consider the process of change and stages of change addressing awareness (e.g., discrepancy between current and healthy behavior), attitudes, self-efficacy (or behavioral control), and social influence (support, pressure, and modeling) as promising concepts for influencing behavior change.

To improve the quality of future research on CTC intervention on F&V intake, it is recommended to report F&V intake data separately, as they are distinct behaviors that can exhibit different responses to CTC intervention. Furthermore, reporting on longer term effects (≥ 12 mo), reaching current recommended guidelines, and tracking and reporting implementation costs would also be advisable. In addition, it would be good to strictly follow CONSORT and TREND recommendations for reporting on randomized and nonrandomized studies.

### Author contributions

The authors’ responsibilities were as follows—IM and AM: designed the review; IM and AM: performed data extraction; AM: performed analysis and led the writing process; WV: provided statistical advice; HV: provided health communication and health behavior models expertise; all authors critically reviewed, read, and approved the final version of the manuscript.

#### Conflicts of interest

The authors report no conflicts of interest.

#### Funding

The authors reported no funding received for this stud*y*.

#### Data availability

Data described in the manuscript, code book, and analytic code will be made available upon request pending.
