# Supplementary material for: Twelve Month Efficacy of Computer-Tailored Communication in Boosting Fruit and Vegetable Consumption Among Adults Aged Forty and over: A Three-Level Meta-Analysis and Systematic Review of Randomized Controlled Trials
Source: Adv Nutr. 2023 Nov 17;15(1):100150. doi: 10.1016/j.advnut.2023.100150 (PMC10756964; doi:10.1016/j.advnut.2023.100150)
Supplement: Multimedia component 1 [file mmc1.docx]

**Supplemental Table 1.** Excluded Studies with Reasons for Exclusion

| **Num.** | **First author, year** | **Reason for Exclusion** |
| --- | --- | --- |
| 1 | Anderson et al., 2001(1) | short follow-up |
| 2 | Baker & Warlde, 2002(2) | short follow-up |
| 3 | Block et al, 2004(3) | short follow-up |
| 4 | Block et al., 2008(4) | protocol |
| 5 | Broekhuizen et al, 2010(5) | protocol |
| 6 | Brug et al., 1996(6) | short follow-up |
| 7 | Brug et al.,1998(7) | short follow-up |
| 8 | Brug et al., 1999(8) | short follow-up |
| 9 | Clark et al, 2002(9) | wrong outcome - SENIOR PROJECT intervention components, results are in Greene et al, 2008 |
| 10 | Delichatsiosis et al., 2001(10) | short follow-up |
| 11 | Demark-Wahnefried el al, 2003(11) | protocol |
| 12 | Elbert et al, 2016(12) | short follow-up |
| 13 | Risica et al., 2009(13) | short follow-up |
| 14 | Gans et al, 2015(14) | short follow-up |
| 15 | Glasgow et al., 2006(15) | short follow-up |
| 16 | Hutchesson et al., 2014(16) | short follow-up |
| 17 | Irvine et al., 2004(17) | short follow-up |
| 18 | Kanera et al., 2016(18) | short follow-up |
| 19 | Ko et al., 2011(19) | wrong outcome – focusing on the mediator variable |
| 20 | Kramish Campbel et al, 1994(20) | short follow-up |
| 21 | Kramish Campbell et al., 1999(21) | multicomponent program with e.g. cooking classes, environmental interventions that should make F&V more assessable, etc.; the main focus of the study impact of the message source on recall and perceived credibility |
| 22 | Kramish Campbell et al., 2002(22) | young population |
| 23 | Kreuter et al., 2005(23) | young population |
| 24 | Kreuter and Strecher, 1996(24) | wrong outcome – focusing on fat intake and other behaviors (F&V consumption was marginally researched and no data on consumption/consumption change available) |
| 25 | Kypri and McAnally, 2005(25) | short follow-up |
| 26 | Luszczynska et at., 2007(26) | short follow-up |
| 27 | Lutz et al., 1999(27) | short follow-up |
| 28 | Moutappa et al., 2011(28) | short follow-up |
| 29 | Mummah et al., 2017(29) | short follow-up |
| 30 | Oenema et al., 2001(30) | wrong outcome - focusing on determinants |
| 31 | Oenema et al., 2005(31) | short follow-up |
| 32 | Oenema and Brug, 2003(32) | wrong outcome - focusing on the intention to change |
| 33 | Parekh et al., 2012(33) | short follow-up |
| 34 | Parekh et al., 2012(34) | protocol |
| 35 | Paxton et al., 2017(35) | short follow-up |
| 36 | Reinwand et al., 2013(36) | protocol |
| 37 | Reinwand et al., 2016(37) | wrong outcome – focus on generating F&V intake action plans |
| 38 | Resnicow et al., 2008(38) | short follow-up |
| 39 | Robroek et al., 2007(39) | protocol |
| 40 | Smeets et al., 2007(40) | short follow-up |
| 41 | Sorensen et al., 2007(41) | short follow-up |
| 42 | Springvloet et al., 2014(42) | protocol |
| 43 | Springvloet et al., 2015(43) | short follow-up |
| 44 | Springvloet et al., 2015(44) | short follow-up |
| 45 | Springvloet et al., 2016(45) | wrong outcome – focusing on mediators |
| 46 | Sternfeld et al., 2009(46) | short follow-up |
| 47 | Storm et al., 2016(47) | short follow-up |
| 48 | Van Keulen et al., 2008(48) | protocol |
| 49 | Van Keulen et al., 2010(49) | wrong outcome – cost effectiveness |
| 50 | Winett et al., 2007(50) | short follow-up |

**Supplemental Table 2.** Study Quality Components and Bias Elements

| **First author, year** | **Sequence Generation** | **Allocation Concealment** | **Clustered Randomi-zation** | **Random-ization after Baseline** | **Baseline Differe-nces** | **Blinding of Partici-pants** | **Blinding Treatment Deliverers** | **Technology Assisted Treatment Delivery (e.g. on-line, on computer)** | **Blinding of Asse-ssors** | **Self-Reported** | **ITT or PP** | **Treatment of Missing Data** | **Attrition** |
| --- | --- | --- | --- | --- | --- | --- | --- | --- | --- | --- | --- | --- | --- |
| **Alexander et al., 2010**(51) | unclear | unclear | NO | YES | YES, but adjusted in the analysis | NO | NA | YES | unclear | YES | PP | unclear | 20.0% |
| **Broekhuizen et al., 2012**(52) | Stratified/comp-uterized procedure (independent researcher) with Microsoft Access. | YES | YES - family members in household clustered to prevent spill over communicati-on and contaminat-ion: allocation was 1:1.1) | YES | YES, but adjusted in the analysis | NO | NO | YES | NA (no in-person contact although no blinding) | YES | ITT and PP | imputed using multiple imputations | 6.6% |
| **Demark-Wahnefried et al., 2007**(53) | Random assignment lists were generated by a project statistician using the software of the Cancer and Leukemia Group B. Block randomization with 8 strata. | YES | NO | YES | NO | maybe | unclear | NO | unclear | YES | ITT | imputed no change in behavior across time for dropouts | >6.7% |
| **Greene et al., 2008**(54) | unclear | unclear | NO | unclear | NO | unclear | unclear | NO | unclear | YES | ITT | the last observation was carried forward | 24.4% |
| **Heimendinger et al., 2005**(55) | unclear | unclear | NO | YES | NO | unclear | unclear | NO | unclear | YES | unclear | unclear | 43% |
| **Jacobs et al., 2011**(56) | Non-stratified randomization technique with a 1:3 versus 2:3 ratio to keep enough power to study dose–response effects. The randomization was performed by hand by an independent person. | YES | NO | unclear | NO | YES/ report blinding | NO | partially | unclear | YES | ITT | the last observation was carried forward | 30.5% |
| **Jones et al., 2003**(57) | Study was a randomized split-plot design with two randomized between-subject factors and one within-subject factor. | unclear | NO | unclear | NO | unclear | unclear | NO | unclear | YES | ITT | unclear | 22.3% |
| **Kanera et al., 2017**(58) | Randomized allocation (ratio of 1:1) was automatically performed using a digital randomizer after the centralized registration of participants. | YES | NO | YES | YES, but adjusted in the analysis | NO | NA (treatment delivered by the computer program) | YES | NA | YES | ITT and PP | imputed based on missing data analysis | 17.5% |
| **Kramish Campbell et al., 2004**(59) | unclear | YES | YES -Churches were clustered, not nearby and some were of different denomina-tions. This was adjusted in the analysis, ICC=0.01 or less. | YES | NO | NO | unclear | NO | unclear | YES | PP | unclear | 28.0% |
| **Kramish Campbell et al., 2009**(60) | unclear | unclear | NO | unclear | NO | NO | unclear | NO | YES | YES | PP | unclear | 10.9% |
| **Kristal et al., 2000**(61) | unclear | unclear | NO | unclear | NO | NO | unclear | NO | unclear | YES | PP | unclear | 13.0% |
| **Parekh et al., 2014**(62) | Permuted block randomization procedure, participants from the same address were randomized into the same group, 2x2 factorial design. | unclear | NO | YES | NO | YES | unclear | NO | unclear | YES | ITT an PP | the last observation was carried forward | 34.6% |
| **Robroek et al., 2012**(63) | Units were randomized by a researcher not involved in the study, based on a table of random numbers (SAS command Ranuni). | YES | YES – randomiza-tion at the worksite level | unclear | YES, but adjusted in the analysis | YES | NO | YES | NA | YES | ITT | the last observation was carried forward | 28.0% |
| **Schulz et al., 2014**(64) | Computer software randomization system. | YES | NO | unclear | YES, but adjusted in the analysis | NO | NA | YES | NA | YES | ITT-implied | missingness at random assumption | 60.6% |
| **Van Keulen et al., 2011**(65) | Stratified computer randomization Actigraph. | YES | NO | YES | NO | NO | NO,  only to collect missing data | NO | NA (no in-person contact although no blinding) | YES | ITT | The primary outcomes were checked for normality. Fruit and PA were square root transformed because of positive skewness. The heteroscedasticity of residuals was checked and not found. Predictors and covariates were checked for multicollinearity which was not found either. | 26%.0 |
| **Van Keulen et al., 2021**(66) |  |  |  |  |  |  |  |  |  |  |  | Participants with the missing outcome for one or more time points were included in the analyses without the imputation of missing values, using the direct likelihood approach. Missing values on covariates were replaced if allowed. |  |
| **Walker et al, 2009**(67) | Quasi-cluster randomization  (randomization of only two geographical areas). | NO | quasi-cluster randomizati-on  (randomi-zation of only two geographical areas) | unclear | NO | YES | unclear | partially YES | unclear | YES | ITT | imputed via the exceptional maximizat-ion (EM) algorithm | >5.0% |
| ITT-intention to treat, ICC-intraclass correlation, NA – not applicable, PP-per protocol | | | | | | | | | | | | | |


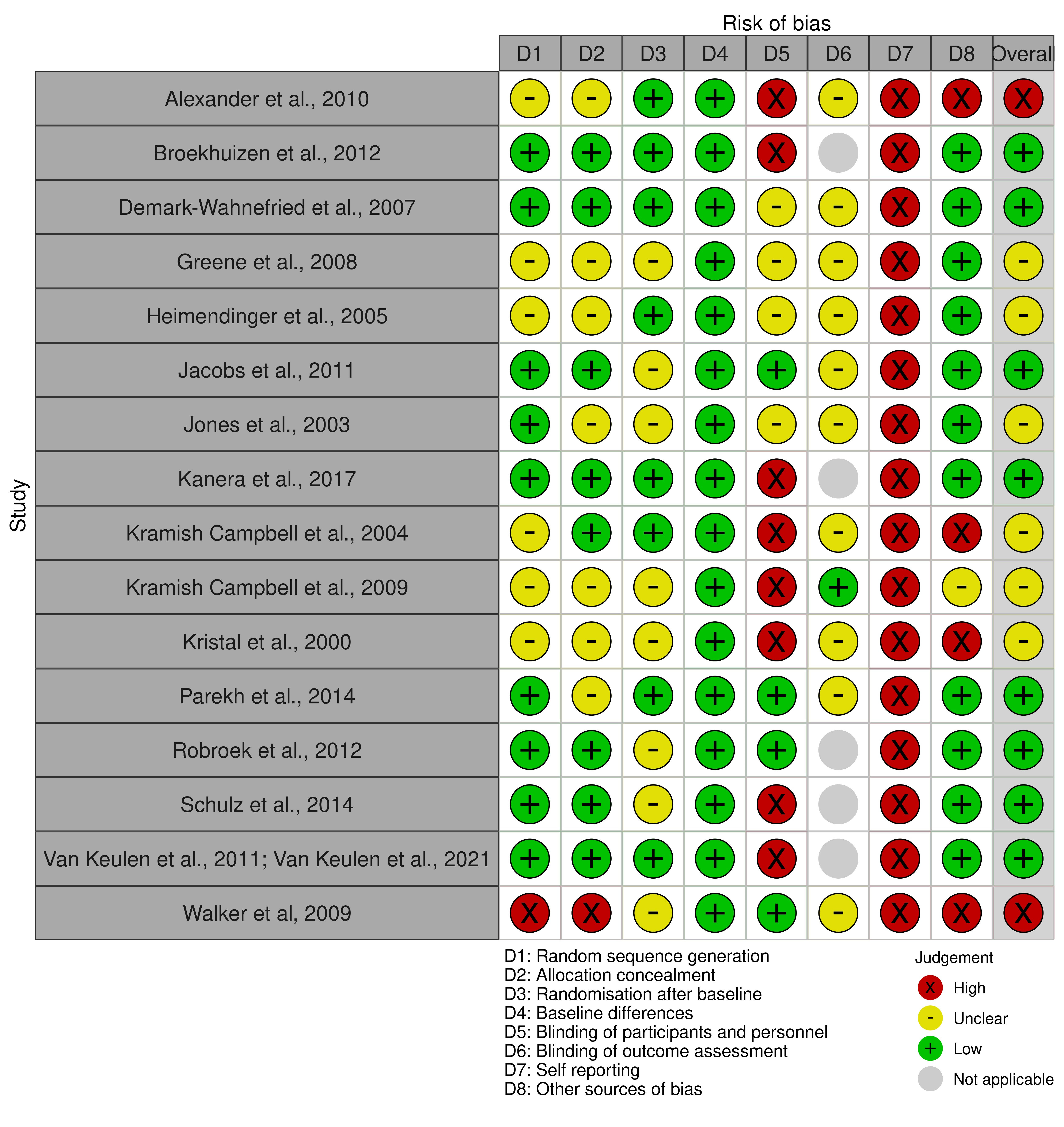


**Supplemental Figure 1**

Individual Study Bias

**Literature**

1. Anderson ES, Winett RA, Wojcik JR, Winett SG, Bowden T. A computerized social cognitive intervention for nutrition behavior: Direct and mediated effects on fat, fiber, fruits, and vegetables, self-efficacy, and outcome expectations among food shoppers. Annals of Behavioral Medicine. 2001;23(2):88–100.

2. Baker A, Biomarkers JWCE and P, 2002 U. Increasing fruit and vegetable intake among adults attending colorectal cancer screening: the efficacy of a brief tailored intervention. AACR [Internet]. 2002 [cited 2021 Dec 22]; Available from: https://cebp.aacrjournals.org/content/11/2/203.short

3. Block G, Wakimoto P, Metz D, Fujii ML, Feldman N, Mandel R, et al. A randomized trial of the little by little cd-rom: Demonstrated effectiveness in increasing fruit and vegetable intake in a low-income population. Prev Chronic Dis. 2004;1(3):1–12.

4. Block G, Sternfeld B, Block CH, Block TJ, Norris J, Hopkins D, et al. Development of Alive! (A Lifestyle Intervention Via Email), and its effect on health-related quality of life, presenteeism, and other behavioral outcomes: randomized controlled trial. J Med Internet Res [Internet]. 2008 [cited 2022 May 10];10(4). Available from: https://pubmed.ncbi.nlm.nih.gov/19019818/

5. Broekhuizen K, Van Poppel MN, Koppes LL, Brug J, Van Mechelen W. A tailored lifestyle intervention to reduce the cardiovascular disease risk of individuals with Familial Hypercholesterolemia (FH): Design of the PRO-FIT randomised controlled trial. BMC Public Health. 2010;10.

6. Brug J, Steenhuis I, Van Assema P, De Vries H. The impact of a computer-tailored nutrition intervention. Prev Med (Baltim). 1996;25(3):236–42.

7. Brug J, Glanz K, Van Assema P, Kok G, Van Breukelen GJP. The Impact of Computer-Tailored Feedback and Iterative Feedback on Fat, Fruit, and Vegetable Intake. Health Education and Behavior. 1998;25(4):517–31.

8. Brug J, Steenhuis I, Van Assema P, Glanz K, De Vries H. Computer-tailored nutrition education: Differences between two interventions. Health Educ Res. 1999;14(2):249–56.

9. Clark PG, Nigg CR, Greene G, Riebe D, Saunders SD. The Study of Exercise and Nutrition in Older Rhode Islanders (SENIOR): Translating theory into research. Health Educ Res. 2002;17(5):552–61.

10. Delichatsios HK, Friedman RH, Glanz K, Tennstedt S, Smigelski C, Pinto BM, et al. Randomized trial of a “talking computer” to improve adults’ eating habits. American Journal of Health Promotion. 2001;15(4):215–24.

11. Demark-Wahnefried W, Clipp EC, McBride C, Lobach DF, Lipkus I, Peterson B, et al. Design of FRESH START: A randomized trial of exercise and diet among cancer survivors. Med Sci Sports Exerc [Internet]. 2003 Mar 1 [cited 2022 Apr 5];35(3):415–24. Available from: https://journals.lww.com/acsm-msse/Fulltext/2003/03000/Design_of_FRESH_START__A_Randomized_Trial_of.6.aspx

12. Elbert S, Dijkstra A, research AOJ of medical I, 2016 undefined. A mobile phone app intervention targeting fruit and vegetable consumption: the efficacy of textual and auditory tailored health information tested in a randomized. jmir.org [Internet]. [cited 2021 Dec 22]; Available from: https://www.jmir.org/2016/6/e147/

13. Risica PM, Strolla LO, Fournier L, Kirtania U, Upegui D, Zhao J, et al. Effectiveness of different methods for delivering tailored nutrition education to low income, ethnically diverse adults. International Journal of Behavioral Nutrition and Physical Activity. 2009 May 5;6.

14. Gans KM, Risica PM, Dulin-Keita A, Mello J, Dawood M, Strolla LO, et al. Innovative video tailoring for dietary change: Final results of the Good for you! cluster randomized trial. International Journal of Behavioral Nutrition and Physical Activity. 2015 Oct 7;12(1).

15. Glasgow RE, Nutting PA, Toobert DJ, King DK, Strycker LA, Jex M, et al. Effects of a brief computer-assisted diabetes self-management intervention on dietary, biological and quality-of-life outcomes. Chronic Illn. 2006 Mar;2(1):27–38.

16. Hutchesson MJ, Collins CE, Morgan PJ, Watson JF, Guest M, Callister R. Changes to dietary intake during a 12-week commercial web-based weight loss program: A randomized controlled trial. Eur J Clin Nutr. 2014;68(1):64–70.

17. Irvine AB, Ary D V., Grove DA, Gilfillan-Morton L. The effectiveness of an interactive multimedia program to influence eating habits. Health Educ Res. 2004;19(3):290–305.

18. Kanera IM, Bolman CAW, Willems RA, Mesters I, Lechner L. Lifestyle-related effects of the web-based Kanker Nazorg Wijzer (Cancer Aftercare Guide) intervention for cancer survivors: a randomized controlled trial. Journal of Cancer Survivorship. 2016 Oct 1;10(5):883–97.

19. Ko LK, Campbell MK, Lewis MA, Earp JA, Devellis B. Information processes mediate the effect of a health communication intervention on fruit and vegetable consumption. J Health Commun. 2011 Mar;16(3):282–99.

20. Campbell MK, DeVellis BM, Strecher VJ, Ammerman AS, DeVellis RF, Sandler RS. Improving dietary behavior: The effectiveness of tailored messages in primary care settings. Am J Public Health. 1994;84(5):783–7.

21. Campbell MK, Bernhardt JM, Waldmiller M, Jackson B, Potenziani D, Weathers B, et al. Varying the message source in computer-tailored nutrition education. Patient Educ Couns. 1999;36(2):157–69.

22. Campbell MK, Tessaro I, De Vellis B, Benedict S, Kelsey K, Belton L, et al. Effects of a tailored health promotion program for female blue-collar workers: Health works for women. Prev Med (Baltim). 2002;34(3):313–23.

23. Kreuter MW, Sugg-Skinner C, Holt CL, Clark EM, Haire-Joshu D, Fu Q, et al. Cultural tailoring for mammography and fruit and vegetable intake among low-income African-American women in urban public health centers. Prev Med (Baltim). 2005;41(1):53–62.

24. Kreuter MW, Strecher VJ. Do tailored behavior change messages enhance the effectiveness of health risk appraisal? Results from a randomized trial. Health Educ Res. 1996;11(1):97–105.

25. Kypri K, McAnally HM. Randomized controlled trial of a web-based primary care intervention for multiple health risk behaviors. Prev Med (Baltim). 2005;41(3–4):761–6.

26. Luszczynska A, Tryburcy M, Schwarzer R. Improving fruit and vegetable consumption: a self-efficacy intervention compared with a combined self-efficacy and planning intervention. Health Educ Res [Internet]. 2007 Oct 1 [cited 2023 Jun 19];22(5):630–8. Available from: https://dx.doi.org/10.1093/her/cyl133

27. Lutz SF, Ammerman AS, Atwood JR, Campbell MK, DeVellis RF, Rosamond WD. Innovative newsletter interventions improve fruit and vegetable consumption in healthy adults. J Am Diet Assoc [Internet]. 1999 Jun [cited 2021 Dec 9];99(6):705–9. Available from: http://www.ncbi.nlm.nih.gov/pubmed/10361533

28. Mouttapa M, Robertson TP, McEligot AJ, Weiss JW, Hoolihan L, Ora A, et al. The Personal Nutrition Planner: A 5-Week, Computer-tailored Intervention for Women. J Nutr Educ Behav [Internet]. 2011 May [cited 2021 Dec 22];43(3):165–72. Available from: https://linkinghub.elsevier.com/retrieve/pii/S1499404610004598

29. Mummah S, Robinson TN, Mathur M, Farzinkhou S, Sutton S, Gardner CD. Effect of a mobile app intervention on vegetable consumption in overweight adults: A randomized controlled trial. International Journal of Behavioral Nutrition and Physical Activity. 2017 Sep 15;14(1).

30. Oenema A, Brug J, Lechner L. Web-based tailored nutrition education: results of a randomized controlled trial. Health Educ Res. 2001;16(6):647–60.

31. Oenema A, Tan F, Brug J. Short-term efficacy of a web-based computer-tailored nutrition intervention: Main effects and mediators. Annals of Behavioral Medicine. 2005;29(1):54–63.

32. Oenema A, Brug J. Feedback strategies to raise awareness of personal dietary intake: Results of a randomized controlled trial. Prev Med (Baltim). 2003 Apr 1;36(4):429–39.

33. Parekh S, Vandelanotte C, King D, Boyle FM. Improving diet, physical activity and other lifestyle behaviours using computer-tailored advice in general practice: a randomised controlled trial. International Journal of Behavioral Nutrition and Physical Activity. 2012 Sep 11;9.

34. Parekh S, Vandelanotte C, King D, Boyle FM. Design and baseline characteristics of the 10 Small Steps Study: A randomised controlled trial of an intervention to promote healthy behaviour using a lifestyle score and personalised feedback. BMC Public Health. 2012;12(1).

35. Paxton RJ, Hajek R, Newcomb P, Dobhal M, Borra S, Taylor WC, et al. A lifestyle intervention via email in minority breast cancer survivors: Randomized parallel-group feasibility study. JMIR Cancer. 2017 Jul 1;3(2).

36. Reinwand D, Kuhlmann T, Wienert J, De Vries H, Lippke S. Designing a theory-and evidence-based tailored eHealth rehabilitation aftercare program in Germany and the Netherlands: Study protocol. BMC Public Health. 2013;13(1).

37. Reinwand DA, Crutzen R, Storm V, Wienert J, Kuhlmann T, De Vries H, et al. Generating and predicting high quality action plans to facilitate physical activity and fruit and vegetable consumption: Results from an experimental arm of a randomised controlled trial. BMC Public Health. 2016 Apr 12;16(1).

38. Resnicow K, Davis RE, Zhang G, Konkel J, Strecher VJ, Shaikh AR, et al. Tailoring a Fruit and Vegetable Intervention on Novel Motivational Constructs: Results of a Randomized Study. Annals of Behavioral Medicine [Internet]. 2008 Apr 10 [cited 2021 Dec 22];35(2):159–69. Available from: https://academic.oup.com/abm/article/35/2/159/4569271

39. Robroek SJW, Bredt FJ, Burdorf A. The (cost-)effectiveness of an individually tailored long-term worksite health promotion programme on physical activity and nutrition: Design of a pragmatic cluster randomised controlled trial. BMC Public Health. 2007;7:1–11.

40. Smeets T, Kremers SPJ, De Vries H, Brug J. Effects of tailored feedback on multiple health behaviors. Annals of Behavioral Medicine [Internet]. 2007 Apr 1 [cited 2023 Jun 19];33(2):117–23. Available from: https://dx.doi.org/10.1007/BF02879892

41. Sorensen G, Barbeau E, … ASCC&, 2007 undefined. Tools for health: the efficacy of a tailored intervention targeted for construction laborers Study1. Springer [Internet]. 2007 Feb [cited 2021 Dec 22];18(1):51–9. Available from: https://link.springer.com/article/10.1007/s10552-006-0076-9

42. Springvloet L, Lechner L, Oenema A. Planned development and evaluation protocol of two versions of a web-based computer-tailored nutrition education intervention aimed at adults, including cognitive and environmental feedback. BMC Public Health [Internet]. 2014 Jan 17 [cited 2021 Dec 9];14(1):47. Available from: http://www.ncbi.nlm.nih.gov/pubmed/24438381

43. Springvloet L, Lechner L, De Vries H, Candel MJJM, Oenema A. Short-and medium-term efficacy of a web-based computer-tailored nutrition education intervention for adultsincluding cognitive and environmental feedback: Randomized controlled trial. J Med Internet Res. 2015 Jan 1;17(1):e23.

44. Springvloet L, Lechner L, De Vries H, Oenema A. Long-term efficacy of a Web-based computer-tailored nutrition education intervention for adults including cognitive and environmental feedback: A randomized controlled trial. BMC Public Health. 2015 Apr 12;15(1).

45. Springvloet L, Lechner L, Candel MJJM, de Vries H, Oenema A. Exploring individual cognitions, self-regulation skills, and environmental-level factors as mediating variables of two versions of a Web-based computer-tailored nutrition education intervention aimed at adults: A randomized controlled trial. Appetite. 2016 Mar 1;98:101–14.

46. Sternfeld B, Block C, Quesenberry CP, Block TJ, Husson G, Norris JC, et al. Improving Diet and Physical Activity with ALIVE. A Worksite Randomized Trial. Am J Prev Med. 2009 Jun;36(6):475–83.

47. Storm V, Dörenkämper J, Reinwand DA, Wienert J, De Vries H, Lippke S. Effectiveness of a web-based computer-tailored multiple-lifestyle intervention for people interested in reducing their cardiovascular risk: A randomized controlled trial. J Med Internet Res. 2016 Apr 1;18(4).

48. Van Keulen HM, Mesters I, Brug J, Ausems M, Campbell M, Resnicow K, et al. Vitalum study design: RCT evaluating the efficacy of tailored print communication and telephone motivational interviewing on multiple health behaviors. BMC Public Health. 2008;8.

49. van Keulen HM, Bosmans JE, van Tulder MW, Severens JL, de Vries H, Brug J, et al. Cost-effectiveness of tailored print communication, telephone motivational interviewing, and a combination of the two: Results of an economic evaluation alongside the Vitalum randomized controlled trial. International Journal of Behavioral Nutrition and Physical Activity [Internet]. 2010 Sep 3 [cited 2023 Jun 19];7(1):1–12. Available from: https://ijbnpa.biomedcentral.com/articles/10.1186/1479-5868-7-64

50. Winett RA, Anderson ES, Wojcik JR, Winett SG, Bowden T. Guide to health: Nutrition and physical activity outcomes of a group-randomized trial of an internet-based intervention in churches. Annals of Behavioral Medicine. 2007;33(3):251–61.

51. Alexander GL, Mcclure JB, Calvi JH, Divine GW, Stopponi MA, Rolnick SJ, et al. A randomized clinical trial evaluating online interventions to improve fruit and vegetable consumption. ajph.aphapublications.org [Internet]. 2010 Feb 1 [cited 2021 Dec 22];100(2):319–26. Available from: https://ajph.aphapublications.org/doi/abs/10.2105/AJPH.2008.154468

52. Broekhuizen K, van Poppel MNM, Koppes LL, Kindt I, Brug J, van Mechelen W. Can Multiple Lifestyle Behaviours Be Improved in People with Familial Hypercholesterolemia? Results of a Parallel Randomised Controlled Trial. PLoS One. 2012 Dec 12;7(12).

53. Demark-Wahnefried W, Clipp EC, Lipkus IM, Lobach D, Snyder DC, Sloane R, et al. Main outcomes of the FRESH START trial: A sequentially tailored, diet and exercise mailed print intervention among breast and prostate cancer survivors. Journal of Clinical Oncology. 2007;25(19):2709–18.

54. Greene GW, Fey-Yensan N, Padula C, Rossi SR, Rossi JS, Clark PG. Change in fruit and vegetable intake over 24 months in older adults: Results of the SENIOR project intervention. Gerontologist [Internet]. 2008 Jun 1 [cited 2021 Dec 20];48(3):378–87. Available from: https://academic.oup.com/gerontologist/article-lookup/doi/10.1093/geront/48.3.378

55. Heimendinger J, O’Neill C, Marcus AC, Wolfe P, Julesburg K, Morra M, et al. Multiple tailored messages are effective in increasing fruit and vegetable consumption among callers to the Cancer Information Service. J Health Commun. 2005;10(SUPPL. 1):65–82.

56. Jacobs N, Clays E, De Bacquer D, De Backer G, Dendale P, Thijs H, et al. Effect of a tailored behavior change program on a composite lifestyle change score: a randomized controlled trial. Health Educ Res [Internet]. 2011 Oct 1;26(5):886–95. Available from: https://academic.oup.com/her/article-lookup/doi/10.1093/her/cyr046

57. Jones H, Edwards L, Vallis TM, Ruggiero L, Rossi SR, Rossi JS, et al. Changes in Diabetes Self-Care Behaviors Make a Difference in Glycemic Control. Diabetes Care [Internet]. 2003 Mar 1;26(3):732–7. Available from: https://diabetesjournals.org/care/article/26/3/732/29152/Changes-in-Diabetes-Self-Care-Behaviors-Make-a

58. Kanera IM, Willems RA, Bolman CAW, Mesters I, Verboon P, Lechner L. Long-term effects of a web-based cancer aftercare intervention on moderate physical activity and vegetable consumption among early cancer survivors: A randomized controlled trial. International Journal of Behavioral Nutrition and Physical Activity. 2017;14(1):1–13.

59. Campbell MK, James A, Hudson MA, Carr C, Jackson E, Oates V, et al. Improving multiple behaviors for colorectal cancer prevention among African American church members. Health Psychology. 2004;23(5):492–502.

60. Campbell MK, Carr C, Devellis B, Switzer B, Biddle A, Ahinee Amamoo M, et al. A Randomized Trial of Tailoring and Motivational Interviewing to Promote Fruit and Vegetable Consumption for Cancer Prevention and Control. Annals of Behavioral Medicine [Internet]. 2009 Oct 1 [cited 2023 Jun 19];38(2):71–85. Available from: https://dx.doi.org/10.1007/s12160-009-9140-5

61. Kristal AR, Curry SJ, Shattuck AL, Feng Z, Li S. A randomized trial of a tailored, self-help dietary intervention: The puget sound eating patterns study. Prev Med (Baltim). 2000;31(4):380–9.

62. Parekh S, King D, Boyle FM, Vandelanotte C. Randomized controlled trial of a computer-tailored multiple health behaviour intervention in general practice: 12-month follow-up results. International Journal of Behavioral Nutrition and Physical Activity. 2014 Mar 19;11(1).

63. Robroek SJW, Polinder S, Bredt FJ, Burdorf A. Cost-effectiveness of a long-term Internet-delivered worksite health promotion programme on physical activity and nutrition: a cluster randomized controlled trial. Health Educ Res [Internet]. 2012 Jun [cited 2023 Jun 19];27(3):399. Available from: /pmc/articles/PMC3337425/

64. Schulz DN, Kremers SPJ, Vandelanotte C, Van Adrichem MJG, Schneider F, Candel MJJM, et al. Effects of a web-based tailored multiple-lifestyle intervention for adults: A two-year randomized controlled trial comparing sequential and simultaneous delivery modes. J Med Internet Res. 2014;16(1).

65. Van Keulen HM, Mesters I, Ausems M, Van Breukelen G, Campbell M, Resnicow K, et al. Tailored print communication and telephone motivational interviewing are equally successful in improving multiple lifestyle behaviors in a randomized controlled trial. Annals of Behavioral Medicine [Internet]. 2011 Feb 1 [cited 2021 May 27];41(1):104–18. Available from: https://academic.oup.com/abm/article/41/1/104/4569538

66. van Keulen HM, van Breukelen G, de Vries H, Brug J, Mesters I. A randomized controlled trial comparing community lifestyle interventions to improve adherence to diet and physical activity recommendations: the VitalUM study. Eur J Epidemiol [Internet]. 2021 Mar 30 [cited 2021 Dec 22];36(3):345–60. Available from: http://link.springer.com/10.1007/s10654-020-00708-2

67. Walker SN, Pullen CH, Boeckner L, Hageman PA, Hertzog M, Oberdorfer MK, et al. Clinical trial of tailored activity and eating newsletters with older rural women. Nurs Res. 2009 Mar;58(2):74–85.
